# Distance from home and working memory: daily associations varying by neighborhood environments in community-dwelling older adults

**DOI:** 10.1007/s10433-025-00841-5

**Published:** 2025-04-05

**Authors:** Minxia Luo, Eun-Kyeong Kim, Robert Weibel, Mike Martin, Christina Röcke

**Affiliations:** 1 University Research Priority Program (URPP) Dynamics of Healthy Aging, University of Zurich, Zurich, Switzerland; 2 Healthy Longevity Center, University of Zurich, Zurich, Switzerland; 3 Department of Psychology, University of Zurich, Zurich, Switzerland; 4 Urban Development and Mobility (UDM), Luxembourg Institute of Socio-Economic Research (LISER), Esch-sur-Alzette, Luxembourg; 5 Department of Geography, University of Zurich, Zurich, Switzerland

**Keywords:** Healthy aging, Cognitive aging, GPS sensor, Out-of-home mobility, Neighborhood walkability

## Abstract

Out-of-home mobility and neighborhood environment have been, respectively, shown to be associated with older adults’ cognitive abilities and they may have combined effects. Adopting an ecological perspective to mobility–cognition associations, this study examined daily maximum distance from home in relation to daily working memory performance in community-dwelling older adults and the moderation effect of neighborhood environments. Analyses included data over 947 days from 109 Swiss older adults aged 65 to 89 years. Over two weeks, participants wore a custom-built mobile GPS tracker and completed a smartphone-based numerical memory updating task seven times per day. Daily maximum distance from home was extracted from the GPS data. Neighborhood environments were assessed with the Neighborhood Environment Walkability Scale at baseline. Multilevel models showed that maximum distance from home was not associated with working memory performance, but there were cross-level moderation effects of neighborhood environments. Daily maximum distance from home was positively (vs. negatively) associated with daily working memory performance in participants who lived in neighborhoods with more (vs. fewer) places for walking and cycling and higher (vs. lower) land use mix-diversity. Out-of-home mobility and neighborhood environment could have combined effects on older adults’ cognitive abilities. Neighborhoods with more places for walking and biking or having a higher mixture of land use could enhance a positive association between traveling a far distance from home and working memory performance.

**Supplementary Information:**

The online version contains supplementary material available at 10.1007/s10433-025-00841-5.

## Introduction

Mobility is an important functional ability to achieve healthy aging, which denotes that older adults can be and do what they have reason to value (World Health Organization 2015). Out-of-home mobility refers to the spatiotemporal patterns of an individual’s movement in their environment outside the home (Fillekes et al. 2019a; Wettstein et al. 2015). Research shows that being mobile out of the home is associated with better cognitive ability and a lower likelihood of cognitive impairment at the interindividual level (Cullen et al. 2022; Wettstein et al. 2014). Further, the neighborhood environment, as an important venue for out-of-home mobility, has been shown to be associated with older adults’ cognitive health (Cerin et al. 2017b; Chen et al. 2022). This study examined whether out-of-home mobility and neighborhood environments were jointly related to cognitive performance in older adults’ daily life.

## Maximum distance from home and cognitive abilities in older age

The exercise of cognitive abilities is believed to be useful for the preservation of abilities, according to the cognitive reserve theory (Scarmeas and Stern 2003), the cognitive enrichment hypothesis (Hertzog et al. 2008), and the “use it or lose it” hypothesis (Bielak 2010). Given its strong link with activity engagement (Gao et al. 2024; Winters et al. 2015), being mobile out of one’s home could foster cognitive stimulation and help preserve cognitive abilities of older adults. Prior research has shown that out-of-home mobility is associated with cognitive functioning in later life (Cullen et al. 2022). For example, spending extensive amounts of time at home is a risk factor for poor cognitive functioning (McCormick et al. 2022). In contrast, older adults who typically spent more time out of home and traveled further distances away from home had higher episodic memory (Wettstein et al. 2014). Older adults who typically traveled further distances away from home were less likely to have severe cognitive impairment (Liddle et al. 2021; Tung et al. 2014).

In particular, while it is assumed that individuals spend most of the time at home and are the most familiar with the home environment (Siła-Nowicka et al. 2016), out-of-home environment may offer opportunities for novelty exposures in exploring the spatial outside world. Novelty exposures could induce neuromodulators, such as norepinephrine and dopamine, which are key to protect against threats and pathology to the aging brain (Düzel et al. 2010; Mather and Harley 2016; Robertson 2013). We considered that the mobility indicator of maximum distance from home, indicating how far an individual travels from their residence, could reflect the level of novelty of the visited area based on distance-based accessibility (Luo et al. 2023a).

In particular, while prior research on maximum distance from home and cognition focused on between-person associations (Liddle et al. 2021; Tung et al. 2014), we underscore the value of focusing on within-person mobility–cognition associations. Examining maximum distance from home varying from one day to the next day within the same person helps reduce the possibility of misinterpreting a habitual trip where an individual regularly travels far away from home to the same place (i.e., a lack of novelty). For example, if a person every day visits the same place far away from home (e.g., 10 km away), this person has an average of 10 km travel distance from home, but their within-person variation regarding this distance is zero. Moreover, out-of-home mobility may have a short-term effect on cognitive performance on a daily basis, considering that research has shown that everyday cognitive and social activities had short-term effects on cognitive performance in older adults over a day (Campbell et al. 2020; Zhaoyang et al. 2021). Additionally, mobility–cognition associations may be bidirectional, such that more distance traveled from home on a day may lead to higher cognitive performance on the next day, and vice versa. It is also conceivable that better working memory on a day leads to a higher likelihood of traveling further distance away from home on that day and on the next day. Within-person data are helpful to determine the temporal ordering of the association examining temporal lag effects. In sum, examining the within-person association between maximum distance from home and cognitive performance over days helps understand whether mobility can enhance cognitive performance of older adults.

## The moderation effects of neighborhood environments on mobility–cognition association

The influence of environment on mobility and cognition is acknowledged by various theories. The healthy aging framework proposed by the World Health Organization illustrates that older adults build and maintain their intrinsic capacity and functional ability through the interaction with their environments (World Health Organization 2015). The ecology theory of aging emphasizes that the environment is key to determine the disparity between older adults’ competence and their adaptive behaviors, denoted by the equation of person-environment interaction P × E (Lawton and Nahemow 1973). Older individuals with declining competence are particularly susceptible to influence from environmental contexts (Wahl and Gerstorf 2020). From the perspective of environmental gerontology, out-of-home mobility links a person (P) with the environment (E) as older adults may face challenges either due to a decline in mobility-related physical and cognitive functioning or environmental limitations to prevent mobility (Penger and Oswald 2017). Thus, the environment is an essential element to be taken into account when understanding mobility–cognition associations.

Neighborhood is an important physical space for older adults’ out-of-home mobility (Wahl and Gerstorf 2018; Webber et al. 2010). Neighborhood is defined by a relatively small area surrounding a person’s home from the perspectives of administrative, geographical, or subjective boundaries (Wörn et al. 2017). It is typically up to 1 km away from home and about a 10–20 min’ walk for older adults without mobility problems (Cerin et al. 2021). Research showed that neighborhood environments were associated with older adults’ out-of-home mobility. For instance, an increase in esthetics (e.g., absence of litter) within 1.2 km from home and an increase of number of destinations per street within 800 m from home were associated with more transportation walking, i.e., walking for the purposes of grocery shopping and visiting family and friends (Etman et al. 2014). Barriers located in 250 to 500 m away from home (e.g.., lack of sidewalks, poor street conditions) were associated with lower physical activity (exercises, transportation activities) (Portegijs et al. 2020).

Further, research showed that neighborhood environments were associated with older adults’ cognitive functioning. Reviews on neighborhood environments and older adults’ cognition revealed that neighborhood characteristics (such as higher land use mix, higher street connectivity, better physical conditions) were associated with higher cognitive abilities and lower dementia risks (Besser et al. 2017; Chen et al. 2022). Neighborhood characteristics reflecting destination accessibility and built environment diversity and quality appear to be closely linked with both out-of-home mobility and cognitive functioning in older age (Cerin et al. 2017a; Song et al. 2024).

An ecological framework of engagement–cognition interactions proposes that environments offer opportunities for activities and experiences and that older adults’ cognition is shaped by how they engage with these opportunities through long-term interactions (Stine-Morrow and Manavbasi 2022). According to this framework, engagement–cognition associations could be enhanced in an enriched ecosystem that offer diverse opportunities to experience and engage and to shape the cognitive system. Viewing mobility as an engagement that is embedded in neighborhood environments, we proposed that the positive association between out-of-home mobility and cognitive performance might be strengthened in an enriched ecosystem, characterized by neighborhoods that are supportive of mobility and benefits cognitive functioning. This research could be translated into knowledge on modifiable environment factors to support preservation of cognitive health in later life (Wahl and Oswald 2010; Zhang et al. 2023).

## The current study

This study examined the association between daily maximum distance from home and daily cognitive performance as a function of neighborhood environments in community-dwelling older adults. First, we examined the association between daily maximum distance from home and daily cognitive performance. Daily cognitive performance was assessed through a smartphone-based working memory task. Working memory is the ability for simultaneous storage and processing of information, and the decline of working memory is a characteristic phenomenon of cognitive aging (Oberauer and Kliegl 2001). Working memory has been shown to be improved through training practices (Li et al. 2008) and activity engagement in older adults’ everyday life (Luo et al. 2023b). We hypothesized that a greater maximum distance from home would be associated with better working memory performance.

Second, we examined whether and how neighborhood environments moderate the association between daily maximum distance from home and daily working memory performance. Neighborhood environment characteristics were assessed through the Neighborhood Environment Walkability Scale (Saelens et al. 2003). This scale was originally developed to assess environments in relation to physical activity and has been broadly used to assess perceived neighborhood environments (Almeida et al. 2021). Although it is under the term of “walkability,” the scale covers various domains that are related to cognition, not only about walkability but also others, such as transportation infrastructure and urban design (Chen et al. 2022). We expected that “more walkable” characteristics of the scale may be supportive of out-of-home mobility and favor cognitive functioning. In turn, associations between out-of-home mobility and cognitive functioning may be enhanced in neighborhoods that were “more walkable.” According to this scale, “more walkable” characteristics refer to a good mix of land use (e.g., a mix of commercial and residential), higher accessibility to services (e.g., stores, parking), adequate walking and biking facilities (e.g., pedestrian trails), good esthetics (e.g., shades), higher safety from traffic and crime, and higher overall satisfaction with neighborhood.^1^

We conducted two follow-up analyses. First, in order to illuminate whether greater mobility on one day predicts better working memory on the next day or vice versa, we examined the temporal ordering of the relation. Second, we examined whether car driving moderated any found associations between mobility, cognition, and neighborhood environment, considering that car driving is closely related to older adults’ mobility, cognition, and neighborhood walkability (Hirsch et al. 2014; Wagner and Nef 2011).

## Methods

We examined data from a large interdisciplinary project on mobility, activity, and social interactions of older adults (Röcke et al. 2023). The study procedures were conducted according to the Declaration of Helsinki and were approved by the Ethics Committee of the Faculty of Arts and Social Sciences of the University of Zurich (permission no. 17.2.4). Written informed consent was obtained from all the participants.

## Participants

Participants were recruited via the lead institute's survey center. Inclusion criteria were age 65 years or above, computer and internet access at home, sufficient vision to use the smartphone, and a score above 26 in the Mini-Mental State Examination. A total of 150 older adults met the study criteria and were compensated with 200 Swiss Francs.

## Study design and procedures

Participants completed questionnaires on sociodemographic information and neighborhood environment at baseline. Across four weeks, participants carried a custom-built mobile sensor (the “uTrail”). Over the first two weeks, participants additionally carried a smartphone with them during their waking hours as they went about their daily lives. They were prompted to complete a smartphone-based working memory task seven times per day (approximately every two hours).

## GPS sensor (“uTrail”)

The uTrail device recorded GPS points every second. The number of GPS recordings varied between participants and across days, due to various factors, such as device malfunction, GPS signal loss, or participants’ non-compliance (Fillekes et al. 2019b). Therefore, as in previous studies (Kim et al. 2021), we established a criterion of including only days that met a minimum recording duration of 8 h to minimize any bias induced by varying GPS recording hours. We extracted a total of 1,720 days from 131 participants who had a valid home location, at least one activity location derived from GPS points, and a minimum of 8-h GPS recordings. Out of the 1,720 days, it was possible to match 931 days (54%) from 109 older adults with the study days for which valid ambulatory working memory assessments existed (*M* = 6.08, *SD* = 2.47). The relatively low matching rate of the data (54%) was caused by the technical reason of non-overlapping observations from two different devices. Simple linear regressions showed no significant differences between participants included versus participants excluded in terms of age, years of education, and income, but slightly more men than women (47%) were included. We consider the data mismatch had limited impact on our results.‬‬‬‬‬‬‬‬‬‬‬‬‬‬‬‬‬‬

The 109 participants aged ranged from 65 to 89 years (*M* = 73.16, *SD* = 5.46, 53% men). About 85% were retired and 58% were married. They had on average 14 years of education (*SD* = 0.5) and income of 4′001 to 6′000 CHF per month. About 39% participants lived in urban, 34% in sub-urban, and 28% in rural areas. On average, participants rated their health  as “very good” and had no depression. About 55% participants drove a car on their own. About 50% reported they used bicycle/e-bike, 47% used tram, 69% used bus, and 84% used train at least once a month.

## Measures

**Daily maximum distance from home.** *Maximum distance from home* referred to the Euclidean distance (kilometers) between a participant's residence and the farthest point visited in a day (Fillekes et al. 2019a). To account for positional uncertainty of GPS points, the spatial extent of ‘home’ was delineated as the area within a radius of 150 m around a valid home location (Fillekes et al. 2019b). It ranged from 0.17 kms to 135.97 kms (*M* = 24.32, *SD* = 24.97) in our study.

**Daily working memory**. Working memory was assessed with two versions of a numerical memory updating task presented on a smartphone (Oberauer and Kliegl 2001; Riediger et al. 2011). Participants were asked to remember four digits between 0 and 9 presented in a 2 × 2 grid and performed several mental operations of addition or subtraction before filling in the final result in each of the four cells. Higher scores (possible range: 0 to 1) indicated greater accuracy and thus better working memory performance. Performance scores were multiplied by 100 for ease of result presentation. Daily working memory was calculated as the average of the working memory scores across a day (*M* = 56.98, *SD* = 16.89, range = 22.09–98.46).

**Neighborhood environments**. The German version of the Neighborhood Environment Walkability Scale (NEWS) included multiple sub-scales to assess different aspects of the neighborhood environment (Bödeker and Bucksch 2011). (a) *Land use mix-diversity*, representing proximity to nonresidential land uses, was assessed by the walking proximity from home to various business or facilities (e.g., supermarket, post office). It ranged from 1–5 min (= 5) to more than 31 min (= 1), with higher scores indicating higher land use mix-diversity in areas closer to home (Cronbach's alpha = 0.88, *M* = 3.00, *SD* = 0.69, range = 1.22–4.39). As follows, the sub-scales were rated on a 4-point scale from 1 (strongly disagree) to 4 (strongly agree), with all items of the sub-scale being averaged and higher scores being better. (b) *Land use mix-access*, representing ease of access to nonresidential uses, was assessed with statements to describe access to services within walking distance, e.g., “I can do most of my shopping at local stores” (Cronbach's alpha = 0.79, *M* = 3.04, *SD* = 0.64, range = 1.14 — 4.00). (c) *Places for walking and cycling* was assessed with statements to describe walking or cycling facilities, such as “The sidewalks in my neighborhood are well maintained” (Cronbach's alpha = 0.71, *M* = 2.87, *SD* = 0.56, range = 1.00 — 4.00). (d) *Esthetics* was assessed with statements to describe neighborhood surroundings, e.g., “My neighborhood is generally free from litter” (Cronbach's alpha = 0.64, *M* = 3.13, *SD* = 0.47, range = 2.00 — 4.00). (e) *Safety from traffic* was assessed with statements to describe pedestrian or traffic safety, e.g., “The speed of traffic on the street I live at is usually slow (30 mph or less)” (Cronbach's alpha = 0.52, *M* = 3.11, *SD* = 0.44, range = 2.00 — 3.88). (f) *Safety from crime* was assessed with statements to describe safety from crime, e.g., “My neighborhood streets are well lit at night” (Cronbach's alpha = 0.52, *M* = 3.45, *SD* = 0.37, range = 2.17–4.00). (g) *Neighborhood satisfaction* was assessed as the average score of satisfaction (1 [strongly dissatisfied] – 5 [strongly satisfied] on various items, such as “the highway access from your home” (Cronbach's alpha = 0.67, *M* = 3.87, *SD* = 0.63, range = 2.06–4.94).

**Covariates**. We controlled for covariates that are known to relate to out-of-home mobility and daily working memory performance (Hirsch et al. 2014; Luo et al. 2023a). That is, *age* in years, *sex* (sex assigned at birth; 0 = women, 1 = men), *years of education, monthly income* (1 = up to 3′000 Fr., 2 = 3′001 to 4′000 Fr., 3 = 4′001 to 6′000 Fr., 4 = 6′001 to 8′000 Fr., 5 = 8′001 to 12′000 Fr., 6 = more than 12′000 Fr.), *retirement status* (0 = non-full-time retired [working part-time / full-time], 1 = full-time retired; 85% retired), and *marital status* (0 = single, divorced, widowed, 1 = married or in long-term relationship; 58% married). *Self-rated health* was assessed with an item from the 12-Item Short-Form Health Survey: “[i]n general, would you say your health is…” (1 = excellent to 5 = poor; *M* = 2.28, *SD* = 0.79) (Ware Jr et al. 1996). *Depression* was assessed using the German version of the Center for Epidemiologic Studies Depression Scale (CES-D) that included 20 items asking participants how often over the past week they experienced symptoms associated with depression (*M* = 7.22, *SD* = 5.17; range = 0–28) (Riediger et al. 1998). A backward digit span task was conducted to assess participants’ baseline working memory, which asked participants to reproduce the same digits in the reverse order (von Aster and Neubauer 2009). It had a total of 7 trials (each trial has 2 sub-trials), starting with 2 digits per trial and increasing up to 7 digits per trials. Each trial can receive a score ranging from 0 to 2, indicating the number of correct sub-trials (M = 6.32, SD = 2.20, range = 0–12). *Car driving* was assessed with a question “[d]o you have the option of using a car in your household?” (1 = yes, I usually drive, 0 = no / yes, but usually others drive). *Residential area* included 0 = urban, 1 = sub-urban, and 2 = rural. In order to account for re-test effects of working memory assessments, we also controlled for *study day*, represented as day since the study began (range = 0–14).

## Analytical approach

Multilevel modeling (Hoffman 2015) was used to examine our research questions and address the hierarchical structure of our data where days were nested in participants. First, we estimated daily working memory as a function of daily maximum distance from home. The model was specified as Eqs. (1)–(3):

1 $$\begin{aligned} & {\text{Level 1}}:{\text{ Working memory}}_{{{\text{ti}}}} \\ & \quad = \, \beta_{{0{\text{i}}}} + \, \beta_{{{\text{1i}}}} ({\text{Maximum distance from home}}_{{{\text{ti}}}}^{ } {-}{\text{Maximum distance from home}}_{{\text{i}}} ) \, + {\text{ e}}_{{{\text{ti}}}} \\ \end{aligned}$$

2 $${\text{Level 2}}: \, \beta_{{0{\text{i}}}} = \, \gamma_{00} + \, \gamma_{{0{1}}} \left( {{\text{Maximum distance from home}}_{{\text{i}}} } \right) \, + \, \gamma_{{0{2}}} \left( {{\text{Moderator}}_{{\text{i}}} } \right) \, + {\text{ u}}_{{0{\text{i}}}}$$

3 $$\beta_{{{\text{1i}}}} = \, \gamma_{{{1}0}} + {\text{ u}}_{{{\text{1i}}}}$$

in which the level-1 model describes within-person variation in working memory via placeholders for the intercept (β_0i_), the slope of within-person variation of maximum distance from home (β_1i_), and a time-specific residual (e_ti_). In the level-2 model, each participant is predicted to have a person-specific intercept (γ_00_ + u_0i_) plus a one-unit difference in between-person variation of maximum distance from home (γ_01_) and a one-unit difference in the moderator (γ_02_), and own slope of within-person variation of maximum distance from home (γ_10_ + u_1i_).

Second, we examined the moderation effects of neighborhood environments (each sub-scale) on the association between daily maximum distance from home and daily working memory performance. The model was specified as Eqs. (1), (4), and (5):

4 $$\begin{aligned} & {\text{Level 2}}: \, \beta_{{0{\text{i}}}} = \, \gamma_{00} + \, \gamma_{{0{1}}} \left( {{\text{Maximum distance from home}}_{{\text{i}}} } \right) \, \\ & \quad + \, \gamma_{{0{2}}} \left( {{\text{Moderator}}_{{\text{i}}} } \right) \, + \, \gamma_{{0{3}}} \left( {{\text{Maximum distance from home}}_{{\text{i}}} } \right)\left( {{\text{Moderator}}_{{\text{i}}} } \right) \, + {\text{ u}}_{{0{\text{i}}}} \\ \end{aligned}$$

5 $$\beta_{{{\text{1i}}}} = \, \gamma_{{{1}0}} + \, \gamma_{{{11}}} \left( {{\text{Moderator}}_{{\text{i}}} } \right) \, + {\text{ u}}_{{{\text{1i}}}}$$

in which level-1 model remains the same as previously described. In the level-2 model, each participant is predicted to have a person-specific intercept (γ_00_ + u_0i_) plus a one-unit difference in between-person variation of maximum distance from home (γ_01_), a one-unit difference in the moderator (γ_02_), and an interaction of between-person variation of maximum distance from home with the moderator (γ_03_). Each participant is also predicted to have one's own slope of within-person variation of maximum distance from home (γ_10_ + u_1i_) plus a cross-level interaction of within-person variation of maximum distance from home with the moderator (γ_11_).

In each model, the covariates were added to estimate the robustness of the findings. Independent variables without a meaningful value of zero were grand-mean centered. Analyses were conducted using the R lme4 package version 1.1–35.1 (Bates et al. 2014) and the R lmerTest package version 3.1–3 (Kuznetsova et al. 2017). Pseudo-R-squared, indicating explained variance, was calculated with the R MuMIn package version 1.47.5 (Barton and Barton 2015). We used the Johnson–Neyman technique through the R Interaction package version 1.1.5 (Long and Long 2019) to determine the regions of the moderator when the mobility–cognition association was significant. Statistical significance was evaluated at *p* < 0.05.

## Results

The intraclass correlation coefficient of maximum distance from home was 0.20 and of working memory was 0.75, suggesting sufficient variance at both the within- and the between-person levels. For the first research question, we examined the association of maximum distance from home with working memory performance. Table 1 shows that maximum distance from home was not significantly associated with daily working memory performance at the within-person or between-person level. Additionally, all sub-scales of neighborhood environments were not associated with working memory performance.

**Table 1:** Associations of maximum distance from home, neighborhood environment, and working memory

Parameter	Base Model			
Fixed effects	Est	SE	Est	SE
Intercept	**42.27**	7.15	**44.85**	7.84
Maximum distance from home (WP)			−0.01	0.01
Maximum distance from home (BP)			0.09	0.06
Land use mix-diversity			2.75	3.08
Land use mix-access			−4.36	3.28
Places for walking and cycling			−2.57	3.33
Esthetics			1.18	3.59
Safety from traffic			3.22	4.30
Safety from crime			−1.62	4.48
Neighborhood satisfaction			−2.76	2.85
Study day	**0.93**	0.08	**0.93**	0.08
Age	−0.31	0.30	−0.31	0.31
Sex	1.46	3.63	1.68	3.83
Years of education	0.87	0.47	0.40	0.54
Retirement status	−8.46	4.27	**−11.12**	4.69
Monthly income	0.56	1.18	0.79	1.26
Marital status	−2.61	3.34	−3.13	3.46
Self-reported health	−2.80	1.93	−3.95	2.16
Depression	−0.14	0.30	−0.05	0.33
Car driving	−0.89	3.15	−2.47	3.40
Residential area	2.15	1.85	1.10	2.12
Backward digit span	**2.71**	0.72	**2.91**	0.76
Random effects (variance)				
Intercept	**199.14**			**205.14**
Slope				**0.004**
Residual	**79.65**			**75.09**
R^2^ (Marginal)	27%			
R^2^ (Conditional)	79%			
Δ R^2^ (Marginal)				2.2%
Δ R^2^ (Conditional)				2.2%

A base model including only covariates is reported for reference. WP = within person; BP = between person; Est. = estimates; SE = standard errors; Δ R^2^ = discrepancy with a base model with covariates only. Boldface correlations are statistically significant (*p* < .05)

**Fig. 1:**
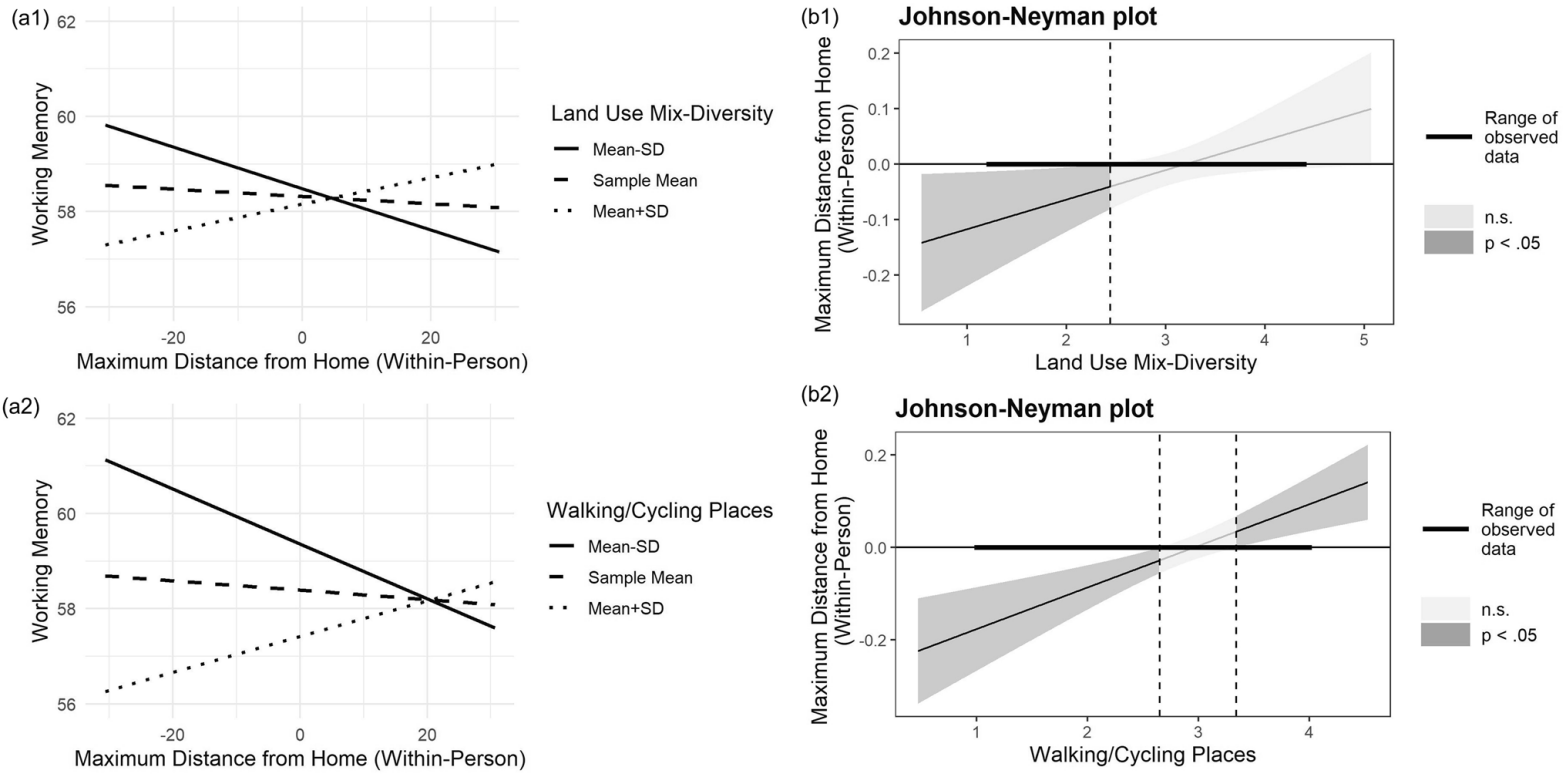
Within-person daily association between maximum distance from home and working memory performances by neighborhood environments. *Note*. Panels a1 and a2 show results from the moderation analysis. Panels b1 and b2 show results from the Johnson–Neyman analysis

For the second research question, we examined whether neighborhood environments influenced the association of maximum distance from home with working memory performance. As seen in Table 2 (Model 1), land use mix-diversity moderated the association between daily maximum distance from home and daily working memory (b = 0.05, SE = 0.02, *p* = 0.033). As shown in Fig. 1 (Panel a1), higher daily maximum distance from home was associated with higher daily working memory in participants who lived in a neighborhood with higher land use mix-diversity, but the association was negative in participants who lived in a neighborhood with lower land use mix-diversity. Analyses with the Johnson–Neyman technique showed that only the negative association in participants with lower land use mix-diversity were observed significant, but the positive association was outside the range of significance in our sample (Fig. 1 panel b1).

**Table 2:** Associations between maximum distance from home and working memory performance by neighborhood environments

Parameter	Working Memory													
Moderators	M1: Land use mix-diversity		M2: Land use mix-access		M3: Places for walking and cycling		M4: Esthetics		M5: Safety from traffic		M6: Safety from crime		M7: Neighborhood satisfaction	
Fixed effects	Est	SE	Est	SE	Est	SE	Est	SE	Est	SE	Est	SE	Est	SE
Intercept	**44.33**	7.56	**46.16**	7.35	**44.72**	7.55	**43.25**	7.31	**43.70**	7.47	**43.77**	7.26	**44.37**	7.27
Distance (WP)	−0.01	0.01	−0.01	0.01	−0.01	0.01	−0.01	0.01	−0.01	0.01	−0.01	0.01	−0.01	0.01
Distance (WP) × Moderator	**0.05**	0.02	0.04	0.02	**0.09**	0.02	0.01	0.03	0.03	0.04	−0.03	0.05	0.03	0.03
Distance (BP)	0.08	0.07	0.10	0.06	0.08	0.07	0.09	0.06	0.08	0.06	0.07	0.06	0.08	0.06
Distance (BP) × Moderator	0.06	0.14	0.09	0.10	0.08	0.12	−0.12	0.15	0.06	0.19	−0.23	0.20	0.01	0.14
Moderator	−0.07	2.69	−3.25	2.58	−1.61	2.91	0.92	3.28	0.31	3.65	−2.39	4.22	−2.92	2.74
Random effects (variance)														
Intercept	**202.57**		**196.84**		**201.08**		**201.21**		**202.34**		**200.60**		**200.19**	
Slope	**0.01**		**0.01**		**0.003**		**0.004**		**0.01**		**0.01**		**0.01**	
Residual	**74.36**		**74.64**		**74.32**		**75.19**		**75.07**		**75.04**		**74.93**	
Δ R^2^ (Marginal)	0.4%		2%		0.9%		0.6%		0.3%		0.4%		1.3%	
Δ R^2^ (Conditional)	1.7%		1.7%		1.6%		1.5%		1.5%		1.4%		1.6%	

All covariates were included but not reported here. Distance = maximum distance from home; WP = within person; BP = between person; Est. = estimates; SE = standard errors; Δ R^2^ = discrepancy with a base model with covariates only. Boldface correlations are statistically significant (*p* < .05)

Similarly, as seen in Table 2 (Model 3) and Fig. 1 (Panel a2), higher daily maximum distance from home was associated with higher working memory performance in participants who lived in a neighborhood with more places for walking and cycling, but the association was negative in participants who lived in a neighborhood with fewer places for walking and cycling (b = 0.09, SE = 0.02, *p* < 0.001). Analyses with the Johnson–Neyman technique showed that the opposing associations between more versus fewer places for walking and biking were both significant (Fig. 1 panel b2). Additionally, we did not find further significant moderation effects of the other dimensions of neighborhood environment characteristics (Table 2).

As a follow-up analysis, we examined the temporal ordering of the mobility–cognition associations, based on Table 2 Models 1 and 3. However, the time-lagged effects were non-significant (Supplementary Table S1). In other words, we could not determine the directionality of the daily mobility–cognition association. Further, we examined whether car driving influenced the neighborhood environment moderator effects on mobility–cognition associations as shown in Table 2 Models 1 and 3. We did not find any significant moderation effects of car driving (Supplementary Table S2). The findings suggest that a neighborhood with higher/lower land use mix-diversity and more/fewer places for walking and cycling might influence both car drivers and non-car drivers equally.

## Discussion

In this study, we examined the association between daily maximum distance from home and daily working memory performance in community-dwelling older adults, and the moderation effects of neighborhood environments. We hypothesized more distance traveled from home would be associated with higher working memory performance. However, we did not find any significant associations at the between- or within- person levels. A prior study with an average of 20.5 days’ (*SD* = 5.9 days) GPS data from community-dwelling older adults found a positive between-person association of distance traveled from home with episodic memory, but not working memory or executive functions (Wettstein et al. 2014). On the one hand, traveling away from home might be less relevant to the numerical working memory task as assessed in this study. On the other hand, participants in our analytical sample had on average 6.08 days’ data (*SD* = 2.47), which may be too short to capture a stable personal characteristic of distance away from home to be associated with individual differences in working memory performance. ‬In our study, only 20% variance of maximum distance from home came from the between-person level, which indicated that out-of-home mobility varied to the largest extent from one day to the next within individuals rather than between persons.^2^

**Fig. 2:**
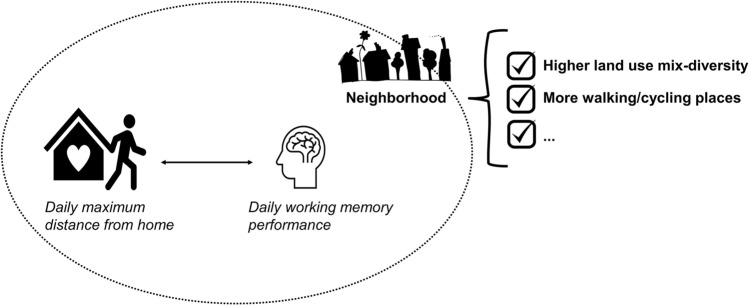
Neighborhood environmental features to influence mobility–cognition associations. *Note*. Our results showed a positive association of daily maximum distance from home with daily working memory performance in older adults living neighborhoods with more places for walking and cycling and higher land use mix-diversity. More modifiable neighborhood environmental features are pending to be discovered

Further, we found that more daily maximum distance from home was associated with higher daily working memory when land use mix-diversity was higher, but the association was negative when land use mix-diversity was lower (Figs. 1 & 2). Markedly, only the negative association in individuals with lower land use mix-diversity was shown significant within the range of land use mix-diversity in our sample. Specifically, the negative association was significant when land use mix-diversity had a score between 1 and 3 (Fig. 1 panel b1), indicating the average distance of business or facilities (e.g., library, gym, pharmacy) were between 20 min and above. Participants in these neighborhoods might travel far away from home to get access to goods and services to satisfy essential needs as daily routines. According to theories that posit the exercise of cognitive abilities as the key to maintain cognitive health (Bielak and Gow 2022; Scarmeas and Stern 2003), traveling away from home for daily routines may not offer novelty exposure and stimulations and may even impoverish stimulation. In contrast, our results suggested that a positive association between maximum distance from home and working memory performance might exist in participants living in neighborhoods with higher land use mix-diversity. This is in line with prior research that showed older adults living in areas with higher land use mix had a positive association with cognitive functioning (Chen et al. 2022; Wu et al. 2015). Further, when these participants travel outside the neighborhood, they could be more likely to get exposure to novel places than those who lived in neighborhoods with lower land use mix-diversity. However, the regions of significance did not fall into our observations. It thus warrants future investigation.

Next, our findings showed that participants who lived in neighborhoods with more places for walking and cycling had a positive association between daily maximum distance from home and daily working memory performance, but the association was negative for participants who lived in neighborhoods with fewer places for walking and cycling (Figs. 1 & 2). Research has indicated that a highly walkable and bikeable neighborhood can increase physical and social activities within the neighborhood area, which in turn benefits cognitive functioning in older adults (Guo et al. 2019; Kim et al. 2023a). Moreover, traveling within a neighborhood with poor physical conditions (e.g., broken sidewalk) requires older adults to pay extra attention to avoid falls (Lee et al. 2018) and this may induce stress, pain, and fatigue (York Cornwell and Goldman 2020). Thus, a more walkable and bikeable neighborhood might promote stepping out of home and traveling beyond the neighborhood and this experience may affect the relation between mobility and cognition.

To date, research has shown that out-of-home mobility and neighborhood environments are, respectively, associated with cognitive functioning in older age (Cullen et al. 2022; Song et al. 2024). Adopting an ecological approach to the mobility–cognition association (Stine-Morrow & Manavbasi 2022), we hypothesized that mobility and neighborhood environment might have combined impact on cognitive functioning in older age. Our findings offer support to the hypothesis. Specifically, more “walkable” neighborhood environment with higher land use mix-diversity and more places for walking and cycling offer enriched opportunities to engage the cognitive muscle and enhance the positive associations between out-of-home mobility and working memory. In contrast, an impoverished ecosystem, such as less “walkable” neighborhoods (i.e., with lower land use mix-diversity and fewer places for walking and cycling), might force individuals to adapt to decreasing cognitive resource use and thus generate negative mobility–cognition associations. In line with recent research and initiatives of developing supportive and empowering environments for older adults with and without cognitive impairments (Finlay et al. 2022; National Academies of Sciences & Medicine, 2024; Seetharaman et al. 2023), our findings offer insights on neighborhood environmental features that could be potentially modified to protect cognitive health in older age as well as support maintenance of functional ability and independence by means of environmental compensation in and for individuals with cognitive impairments including dementia.

That said, it is noteworthy that the effect size of the found moderation effects of neighborhood environments was small (i.e., the increased Pseudo-R-squared compared with the base model in Table 2). Moreover, other aspects of neighborhood environments, such as esthetics and safety from crime, were unrelated to the day-to-day mobility–cognition relations. We conceive several possible explanations. First, we noted that our participants did not rate the lowest levels of several sub-scales of the neighborhood environment measures, indicating that neighborhood environments were relatively “walkable” to our participants. The lack of variance might have explained the non-significant findings. Further, it is shown that older adults spent only part of their time inside their neighborhood (Prins et al. 2014; York Cornwell and Cagney 2017). Similarly, our data indicated that only 15% of the days included maximum distance from home shorter than 1 km, which is the distance the neighborhood is typically defined with (Cerin et al. 2021). Depending on the degree of similarity between residential versus nonresidential environments (Chaix et al. 2017), environments outside the neighborhood could also have an impact on mobility and cognition. Nevertheless, neighborhood boundaries, which do not necessarily have a clear-cut limit at 1 km, can vary based on various physical (e.g., street networks, barriers, modes of travel) and social factors (e.g., activities locations) (Boruff et al. 2012; Hirsch et al. 2014; Pinchak et al. 2021).

More importantly, beyond the classical conception of person × environment interaction, several modern theories in environmental gerontology have illustrated more complex dynamics on how environmental contexts could influence aging processes (Oswald et al. 2024). For example, taking an agency and belonging approach, agency refers to goal-directed behaviors that make use of objective environment characteristics and affordances, whereas belonging refers to non-goal-oriented cognitive and emotional evaluations and interpretations of a space (Chaudhury and Oswald 2019). Our findings of walkability of neighborhood environments covers the agency aspect, but we did not address the belonging aspect of environmental contexts, such as attachment and bonding to the neighborhood, which could potentially also influence mobility–cognition associations. Further, the framework of COntext Dynamics in Aging (CODA) proposes that environmental contexts do not only include the physical dimension of neighborhood structural and infrastructural conditions, but also include other dimensions, such as socioeconomic, social, care and service, and technology (Wahl and Gerstorf 2020). That is, apart from environments inside and outside the neighborhood, other spaces, such as close relationships (social) and wealth (socioeconomic) should also be considered in the investigation of mobility–cognition associations. Additionally, from the perspective of person × environment interaction, out-of-home mobility is likely not only associated with cognition, but also with personal characteristics, such as behavioral flexibility and individual preference for routines (Penger and Oswald 2017). All in all, our study represents an initial attempt to understand the role of environmental contexts in helping to explain some of the observed heterogeneity in the association of mobility and cognition. Further evidence is needed for a more detailed understanding of the role of environmental contexts and their value in cognitive aging research.

## Limitations and future directions

This study incorporated GPS data and ambulatory cognitive assessment to examine within-person associations between daily maximum distance from home and working memory performance in relation to neighborhood environments. Its results highlighted the importance of environmental contexts in mobility–cognition associations. We acknowledge several limitations for interpretation of our results. First, operational definitions of the key constructs (mobility, cognition, and neighborhood environments) could be improved or diversified, so as to gather more comprehensive evidence on the phenomenon investigated. Specifically, mobility can be characterized by other dimensions, temporally and spatially (Fillekes et al. 2019a; Wettstein et al. 2014). Future research could also consider other mobility indicators and may discover additional associations with cognitive performance. Relatedly, we examined as it is closely related to other cognitive abilities (Bartsch and Shepherdson 2022), but future research could investigate more cognitive domains (Giannouli et al. 2018). Additionally, there were other aspects of neighborhood environments to be considered, such as services facilities (e.g., education or cultural facilities) that offer opportunities for social interactions and cognitive stimulations (Zhang et al. 2023) and subjective evaluation of physical and social aspects of neighborhood, such as neighborhood social cohesion (Zhang et al. 2019) and neighborhood stressors (Muñoz et al. 2020). Future research could also incorporate different approaches—combining self-report and sensor-based assessments of neighborhood environments—and examine their relations with mobility and cognition (Lee and Waite 2018; Ng et al. 2018).

Second, our follow-up analysis showed that car driving did not influence the findings, but modes of travel could influence distance traveled from home and the size of activity space (Hirsch et al. 2014) and be associated with cognition (Wagner and Nef 2011). Our findings might reflect the excellent public transportation infrastructure in Switzerland that often makes car ownership less needed, particularly in urban contexts. Future research could still take into account influence of mode of travel, as well as other correlates, such as trip-based behavioral (e.g., trip purposes such as instrumental versus recreational) and environmental characteristics (e.g., trip destinations) (Gao et al. 2024; Kim et al. 2023b; Mou et al. 2024). This will enable more precise reasoning on mechanistic relationships between mobility, environment, and working memory performance.

Third, our findings regarding the temporal ordering of daily mobility–cognition association were inconclusive. Activity enrichment effects on cognitive performance could occur within hours or minutes (Luo et al. 2023c; Olivo et al. 2021). Future research could consider examining data at a higher temporal resolution than a daily aggregation. Moreover, prior research has shown that cognitive impairment was a determinant of reduced mobility of older adults (Ullrich et al. 2019, 2021). Effects of cognition on mobility might thus be more evident in older adults who have pronounced cognitive decline that begin to influence their functional ability and goal-directed behaviors. Such hypothesis is in line with the notion of the cognitive reserve hypothesis that posits compensation could be best observed in vulnerable individuals with clinically relevant decline (Stern 2012), but it warrants further investigation. Relatedly, our sample focused on community-dwelling older adults who were active and highly functioning in Switzerland. To examine the generalizability of our findings, future studies could include participants with more heterogenous cognitive capacities, sociodemographic backgrounds, and from other countries that have different geographical landscapes (Song et al. 2024).

## Conclusion

This study used a combination of GPS sensor and ambulatory cognitive assessment to study mobility and cognitive performance in daily life, taking into account the moderation effects of interindividual differences in neighborhood environment characteristics. Adopting an ecological approach to the mobility–cognition association (Stine-Morrow and Manavbasi 2022), our study offers an empirical example of how environmental resources could be adapted and improved to maintain and enhance older adults’ cognitive performance. Specifically, our findings suggest that it may be useful for preserving cognitive health by building neighborhoods with a high mixture of land use and with more places for walking and cycling and encouraging older adults to be mobile out of home and travel further away from home. Taken together, our findings can be regarded as a stepping stone for the neighborhood environment design considerations that aim to preserve cognitive health in later life.

## Supplementary Information

Below is the link to the electronic supplementary material.

Supplementary file1 (DOCX 22 KB)

## Data Availability

The data that support the findings of this study are not publicly available because the data belong to an ongoing longitudinal study. The data are available upon reasonable request from the data sharing committee via Dr. Christina Röcke (christina.roecke@uzh.ch).

## Footnotes

1 Two sub-scales were dropped because of low reliability: Residential density (Cronbach's alpha = 0.07) and street connectivity (Cronbach's alpha = 0.35).

2 Notably, the variance of maximum distance from home at the between-person level was much smaller (i.e., 5%) in participants who were not car drivers, suggesting that non-car drivers had much more variance in mobility across days at the within-person level than car drivers.
