# Supplementary material for: Distance from home and working memory: daily associations varying by neighborhood environments in community-dwelling older adults
Source: Eur J Ageing. 2025 Apr 5;22(1):17. doi: 10.1007/s10433-025-00841-5 (PMC11972263; doi:10.1007/s10433-025-00841-5)
Supplement: Supplementary file 1 — Supplementary file1 (DOCX 22 KB) [file 10433_2025_841_MOESM1_ESM.docx]

**Supplementary Materials**

Table S1: Follow-Up Analysis of Mobility-Cognition Lead-Lag Associations

| Outcomes | Working Memory | | | | Maximum Distance from Home | | | |
| --- | --- | --- | --- | --- | --- | --- | --- | --- |
| Moderators | M1: Land Use Mix-Diversity | | M2: Places for Walking and Cycling | | M3: Land Use Mix-Diversity | | M4: Places for Walking and Cycling | |
| Fixed effects | Est. | SE | Est. | SE | Est. | SE | Est. | SE |
| Intercept | **41.36** | **8.26** | -6.78 | 10.48 | **44.29** | **8.36** | -6.34 | 10.66 |
| Predictor_t_ (WP) | **-0.02** | **0.01** | -0.22 | 0.15 | -0.01 | 0.01 | -0.22 | 0.15 |
| Predictor_t-1_ (WP) | -0.00 | 0.01 | 0.07 | 0.18 | -0.00 | 0.01 | 0.07 | 0.18 |
| Predictor_t_ (WP) × Moderator | 0.02 | 0.02 | 0.17 | 0.20 | **0.06** | **0.03** | 0.38 | 0.26 |
| Predictor_t-1_ (WP) × Moderator | 0.01 | 0.02 | -0.19 | 0.25 | 0.01 | 0.02 | -0.47 | 0.33 |
| Random effects (variance) | | |  |  |  |  |  |  |
| Intercept | **222.55** |  | **202.45** |  | **218.94** |  | **202.48** |  |
| Slope | 0.000 |  | **0.61** |  | **0.002** |  | **0.59** |  |
| Residual | **63.71** |  | **883.03** |  | **62.48** |  | **880.68** |  |

*Note*. All covariates were included but not reported here. WP = within person; Est. = estimates; SE = standard errors.

Boldface correlations are statistically significant (*p* < .05).

|  | Working Memory | | | |
| --- | --- | --- | --- | --- |
| Moderators | M1: Land Use Mix-Diversity | | M2: Places for Walking and Cycling | |
| Fixed effects | Est. | SE | Est. | SE |
| Intercept | **43.88** | 7.61 | **44.73** | 7.59 |
| Maximum distance from home_t_ (WP) | -0.02 | 0.03 | -0.04 | 0.02 |
| Maximum distance from home_t_ (WP) × Moderator | 0.07 | 0.04 | **0.13** | 0.04 |
| Maximum distance from home (WP) × Moderator × Car driving | -0.02 | 0.05 | -0.04 | 0.05 |
| Random effects (variance) | | |  |  |
| Intercept | **203.59** |  | **203.31** |  |
| Slope | **0.01** |  | **0.003** |  |
| Residual | **74.39** |  | **74.36** |  |

Table S2. Follow-Up Analysis of Moderator Effect of Car Driving

*Note*. All covariates were included but not reported here. WP = within person; Est. = estimates; SE = standard errors.

Boldface correlations are statistically significant (*p* < .05).
